# Calcium antagonism effects on cerebral blood flow in rats with acute hydrocephalus

**DOI:** 10.1186/2045-8118-12-S1-P49

**Published:** 2015-09-18

**Authors:** Alexander Victor Shulyakov, Richard J Buist, Domenico L DiCurzio, Marc Ronald Del Bigio

**Affiliations:** 1 Department of Pathology University of Manitoba, Canada; 2 Manitoba Institute of Child Health, Winnipeg, Canada; 3 Department of Radiology, Faculty of Medicine University of Manitoba, Canada; 4 Department of Human Anatomy & Cell Science University of Manitoba, Canada

## Introduction

Calcium ion antagonism shows some benefit in experimental hydrocephalus, but the interrelationship between the cerebral vasculature, cerebral blood flow (CBF), and ventriculomegaly remains unclear.

## Methods

Rats were injected with kaolin at 35 days and were studied after 12-15 days. CBF was measured with and without nimodipine or magnesium sulfate using magnetic resonance and fluorescent microspheres. Arterial blood and intracranial pressure (ICP), acid-base, oxygen/hemoglobin, and electrolytes status was measured. Intraparenchymal vascular remodeling was also determined in brains of hydrocephalic rats treated with calcium antagonists for 2 weeks.

## Results

Increased ICP did not significantly affect cerebral perfusion pressure within the range of normal autoregulation. CBF significantly decreased in acutely hydrocephalic rats. Nimodipine and magnesium sulfate decreased systemic arterial blood pressure, cerebral perfusion and intracerebral pulse pressure; however, there was no change in cerebral blood flow. There was no change in white matter vascular density after 2 weeks treatment.

## Conclusions

Cerebral hypoperfusion occurs in acute experimental hydrocephalus, however the calcium channel antagonists nimodipine and magnesium sulfate, do not increase the CBF. Reduced intracranial pulse pressure possibly mitigates development of acute hydrocephalus.
